# Dehiscence and fenestration of Class I individuals with normality patterns in the anterior region: a CBCT study

**DOI:** 10.1007/s00784-022-04384-2

**Published:** 2022-03-07

**Authors:** Liangyan Sun, Chuangchuang Mu, Li Chen, Bingjiao Zhao, Jie Pan, Yuehua Liu

**Affiliations:** Department of Orthodontics, Shanghai Stomatological Hospital & School of Stomatology, Fudan University, No.356 East Beijing Road, Shanghai, China

**Keywords:** Normal occlusion, Dehiscence, Fenestration, CBCT

## Abstract

**Objectives:**

The purpose of this study was to investigate the prevalence of alveolar bone dehiscence and fenestration of Class I individuals with normality patterns in the anterior region using cone-beam computed tomography (CBCT).

**Materials and methods:**

A total of 4715 retrospective cases from January 2018 to December 2020 in the Orthodontic Department of Shanghai Stomatological Hospital were screened. Sixty-one cases were Class I individuals with normality patterns in the anterior region. Their incidence of dehiscence and fenestration in the anterior teeth region was studied and statistically analyzed.

**Results:**

Dehiscence was found in 27.46% of the evaluated anterior teeth and fenestration was found in 26.91% of anterior teeth. Severe dehiscences and fenestrations mainly occurred in mandibular canines and maxillary canines, respectively. Alveolar bone defects were present in 100% of patients, while one patient had alveolar bone defects in 91.67% of the anterior teeth.

**Conclusions:**

Dehiscence was found in 27.46% of the anterior teeth of Class I individuals with normality patterns, while fenestration was found in 26.91% of them. Alveolar bone defects were present in 100% of patients.

**Clinical relevance:**

Alveolar bone dehiscence and fenestration were normal and common in our sample, indicating that they are more likely to be physiological rather than pathological defects. Orthodontists should be aware of the presence and severity of these defects before treatment in order to avoid both possible complications and overtreatment.

## Introduction

Healthy alveolar bone is a basic requirement for orthodontic treatment, while the inadequate height and thickness of alveolar bone, or defects, make orthodontic treatment difficult. Vertical alveolar bone defects are classified as alveolar bone dehiscence and fenestration. In the 1970s, alveolar bone dehiscence and fenestration were of interest to the academic community [1]. Currently, it is generally accepted that alveolar bone dehiscence is a V-shaped defect from the crown to the root of the tooth, involving the apex of the alveolar ridge [1, 2]. Alveolar bone fenestration is an alveolar bone defect that does not involve the apex of the alveolar ridge and usually occurs in the middle or apical 1/3 of the root [1, 2]. Both defects result in localized root exposure.

Prior studies in dry skulls revealed that alveolar bone dehiscence and fenestration were prevalent among various ethnic groups with different types of malocclusions [1–10]. The prevalence of bone dehiscence ranges from 0.99 to 13.4%, while the prevalence of fenestration ranges from 0.23 to 16.9% [11]. Studies have shown that alveolar bone dehiscence was closely associated with gingival recession [12] and that orthodontic treatment in the presence of alveolar bone deficiency may cause increased periodontal damage [13–15]. Therefore, it is essential to assess the root-bone relationship before treatment and to diagnose potential alveolar bone defects to avoid potential treatment risks. Traditional imaging methods cannot accurately detect alveolar bone dehiscence and fenestration, while cone-beam computed tomography (CBCT) exhibits high accuracy and reliability in detecting such defects [16–18].

In recent years, scholars have studied the prevalence of alveolar bone dehiscence and fenestration using CBCT. In 2010, Evangelista K et al. [11] studied 79 Class I and 80 Class II Division 1 malocclusion patients, including a total of 4319 teeth, and found that the prevalence of alveolar bone dehiscence and fenestration was 51.09% and 36.51%, respectively. In 2012, Yagci A et al. [19] studied 41 Class I, 42 Class II, and 40 Class III patients, and found that alveolar bone dehiscence had the highest prevalence in the lower jaw of Class III patients (42.64%), while alveolar bone fenestration had the highest prevalence in the upper jaw of Class II patients (19.49%). In 2013, Sun L et al. [20] studied 44 cases of skeletal Class III malocclusion and found that the prevalence of alveolar bone dehiscence and fenestration was 61.57% and 31.93%, respectively, in the lower anterior region. Zhou L et al. [21] studied 587 upper and lower anterior teeth of 50 patients with bimaxillary protrusion and found that 224 of which had alveolar bone defects (38.16%). Thus, the results of those studies suggested that alveolar bone dehiscence and fenestration were common among all types of malocclusions. However, the prevalence of alveolar bone dehiscence and fenestration in cases of normal occlusion has not been studied. Moreover, it is unknown if such defects are normal and common in the population, or if they are closely related to the occurrence of malocclusion. Thus, the aim of this study was to investigate the prevalence of alveolar bone dehiscence and fenestration in the anterior region of Class I individuals with normality patterns using CBCT.

## Materials and methods

### Subjects and samples

A total of 4715 retrospective cases from January 2018 to December 2020 in the Orthodontic Department of Shanghai Stomatological Hospital were screened, and Class I individuals with normality patterns in the anterior region were selected. The upper and lower anterior teeth of such individuals were the study subjects. The inclusion criteria were: (1) bilateral Class I molar and canine relationships; (2) ANB was greater than 0°, but less than 3°; (3) normal overjet and overbite on anterior and posterior teeth; (4) aligned anterior teeth without obvious crowding (less than 2 mm) and no obvious spacing between teeth (less than 0.5 mm); (5) Spee curve depth of less than 2 mm; and (6) no obvious rotation (less than 5°) in the anterior region. The exclusion criteria were: (1) obvious wear; (2) defective dentition or supernumerary teeth; (3) periodontal disease with significant probing depth (PD > 3 mm) or attachment loss (AL > 0 mm), or with the crest of the interproximal bone more than 2 mm apical from the cemento-enamel junction of the correspondent tooth; (4) patients with a history of restorations, orthodontics, or maxillofacial surgery; and (5) craniofacial syndromes or obvious pathologies. According to the above criteria, 61 Class I individuals with normality patterns in the anterior region were included in this study, including 32 males and 29 females aged 17–23 years (mean age of 19.8 years). A total of 732 upper and lower anterior teeth were included as subjects. The distribution is shown in Table 1. The study was approved by the independent Ethics Committee of Shanghai Stomatological Hospital (certificate number 2021–001). All participants were informed that their CBCT data were to be used in this study and they provided signed informed consent.

**Table 1:** Distribution of teeth examined by tooth type in 32 male and 29 female subjects

	Male	Female	
	Subjects	Subjects	Total
Maxillary central incisor	64	58	122
Maxillary lateral incisor	64	58	122
Maxillary canine	64	58	122
Mandibular central incisor	64	58	122
Mandibular lateral incisor	64	58	122
Mandibular canine	64	58	122
Total	384	348	732

### Acquisition of CBCT data

**Fig. 1:**
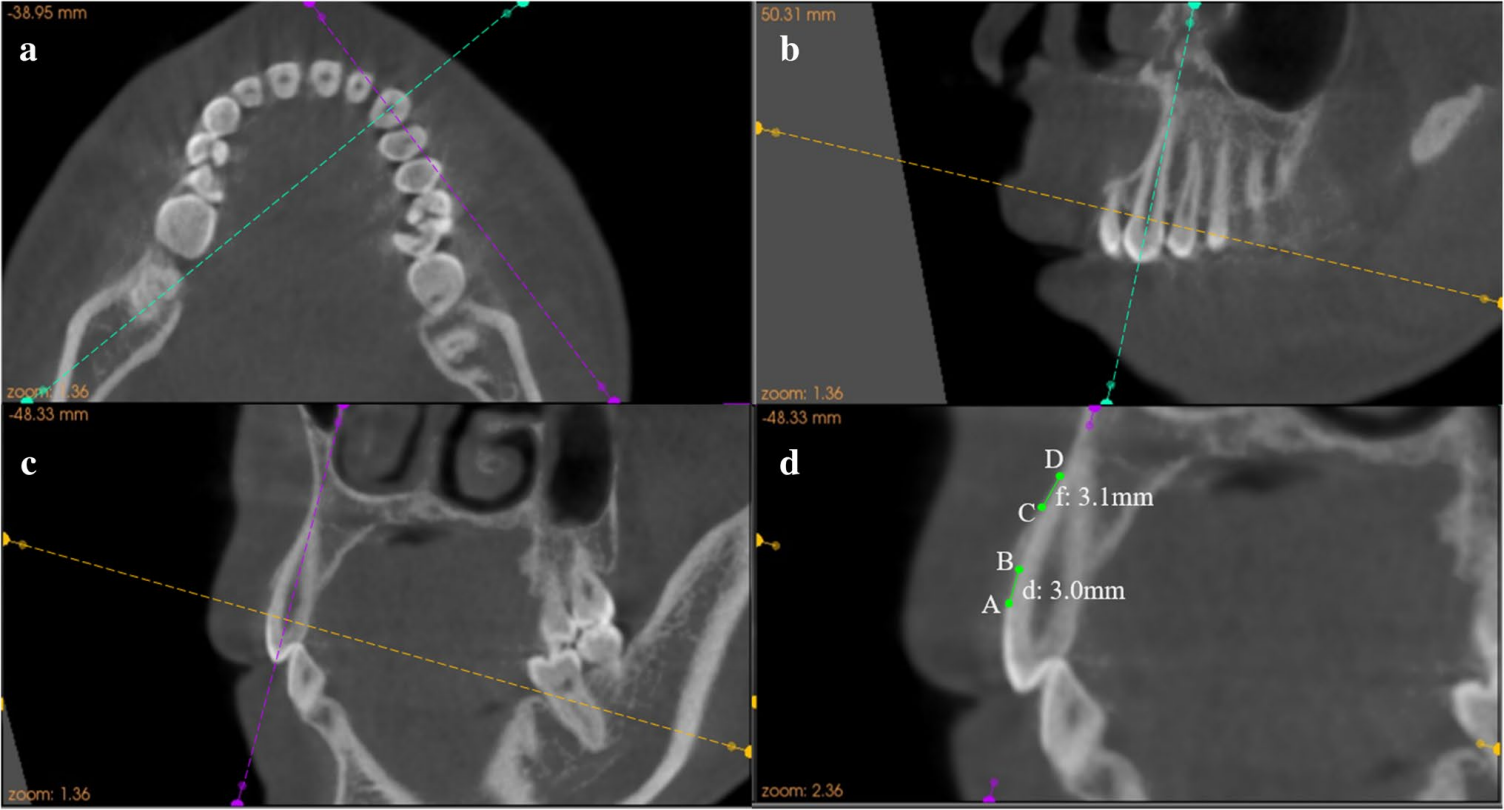
Illustration of the measurement plane, reference points, and variables. **a** By adjusting the horizontal view to the maximum root cross-section, the sagittal plane (shown by the green line) passed through the most convex point of its labiolingual surface. **b** The moving sagittal plane was turned in the coronal view (shown by the green line) to pass through the cusp of the tooth with the root tip. **c** The coronal plane was turned in the sagittal view (shown by the purple line) to pass through the cusp of the tooth with the root tip. **d** The abovementioned steps were repeated with fine adjustments each time, at which point the sagittal section of the tooth was the largest labiolingual section. Both dehiscence (3.0 mm) and fenestration (3.1 mm) were detected in the selected maxillary canine. The definition of reference points and variables is shown in Table 2

CBCT images (KaVo, Germany) were obtained as part of the routine examination before the orthodontic diagnosis and treatment planning. After the CT instrument’s calibration was ascertained to be correct, the patient’s head was oriented by locating the Frankfort plane parallel to the horizontal plane and in centric occlusion. The scanning parameters were voxel size, 0.25 mm; FOV, 170 mm; voltage, 120 kV; current, 5 mA; and scan time, 17.8 s. The DICOM data obtained from the CBCT scans from the 61 cases were reconstructed in three dimensions using image analysis software (Kodak Dental Imaging Software 3D Module V2.4, Eastman Kodak, Rochester, NY, USA). Referring to the method of Sun L et al. [18, 20], the largest labiolingual section of the sample incisors and canines was selected as the measurement plane for alveolar bone dehiscence and fenestration (as shown in Fig. 1). The reference points and measurement variables were the same as in a previous study [22] and shown in Table 2. The *d* and *f* values of the 732 upper and lower anterior teeth were calculated and recorded. If there was no fenestration on the measurement plane, the *f* value was recorded as 0 mm. If the *d* value was greater than 2 mm, the tooth was regarded as exhibiting alveolar bone dehiscence [11, 18]. If the *f* value was greater than 2.2 mm, the tooth was regarded as exhibiting alveolar bone fenestration [18]. All measurements were made by the same operator (L.S.) and were re-measured 4 weeks after the initial measurements. The mean values of the first and second measurement results were then used for analysis.

**Table 2:** Definition of reference points and variables

Reference points and variables	Definition
Dehiscence	Alveolar bone defect involving an alveolar margin 2 mm or greater and concurrent with a V-shaped bone margin
Fenestration	A circumscribed defect on the alveolar bone exposing the root, not involving the alveolar crest
A	CEJ at the labial side
B	Alveolar crest at the labial side
C	The coronal border of a fenestration
D	The apical border of a fenestration
d (mm)	The distance between A and B
f (mm)	The distance between C and D

### Statistical analysis

The prevalence and distribution of alveolar bone dehiscence and fenestration on the labial side of the upper and lower anterior teeth were determined. Alveolar bone dehiscence and fenestration were graded according to the *d* and *f* values, respectively. A 2 < *d* ≤ 4 mm was considered mild alveolar bone dehiscence; a 4 < *d* ≤ 6 mm was considered moderate dehiscence; and a *d* > 6 mm was considered severe dehiscence. A 2.2 < *f* ≤ 4 mm was considered mild fenestration; a 4 < *f* ≤ 6 mm was considered moderate fenestration; and an *f* > 6 mm was considered severe fenestration.

The distributions of the severity of alveolar bone dehiscence and fenestration were determined separately, and the number of bone dehiscence and fenestration present in the anterior teeth of each case was calculated.

Statistical analyses were performed using the SPSS 23.0 statistical package. Descriptive statistics are used to analyze the presence of dehiscence and fenestration in different teeth, as well as their correlation. Intraclass correlation coefficients (ICCs) were calculated to assess the intra-observer agreement. ICC estimates and their 95% confident intervals were calculated based on a single-rating ((intra-agreement)/mean-rating (inter-agreement)), absolute-agreement, 2-way mixed-effects model.

## Results

The prevalence of alveolar bone dehiscence and fenestration in the included samples is shown in Table 3. Among the 732 upper and lower anterior teeth, the prevalence of alveolar bone dehiscence was 27.46%, the presence of fenestration was 26.91%, and the presence of alveolar bone defects was 48.91%. The prevalence of dehiscence was highest in mandibular canines (44.26%), followed by maxillary canines (28.69%), and lowest in the maxillary central incisors (9.84%). The prevalence of fenestration was highest in maxillary lateral incisors (56.56%), followed by the maxillary canines (44.26%), and lowest in mandibular central incisors (4.92%). Bone defects were highest in maxillary lateral incisors (71.31%) and lowest in maxillary central incisors (16.39%).

**Table 3:** Prevalence of different types of defects in the anterior region

Tooth type	Dehiscence		Fenestration		Either defect		Both defects	
	*n*	%	*n*	%	*n*	%	*n*	%
Maxillary central incisor	12	9.84	9	7.38	20	16.39	1	0.82
Maxillary lateral incisor	35	28.69	69	56.56	87	71.31	17	13.93
Maxillary canine	38	31.15	54	44.26	78	63.93	14	11.48
Upper anterior region	85	23.22	132	36.07	185	50.55	32	8.74
Mandibular central incisor	26	21.31	6	4.92	32	26.23	0	0
Mandibular lateral incisor	36	29.51	28	22.95	59	48.36	5	4.10
Mandibular canine	54	44.26	31	25.41	82	67.21	3	2.46
Lower anterior region	116	31.69	65	17.76	173	47.27	8	2.19
Total	201	27.46	197	26.91	358	48.91	40	5.46

^*^Either defect: dehiscence or fenestration. *Both defects: dehiscence combined with fenestration

**Fig. 2:**
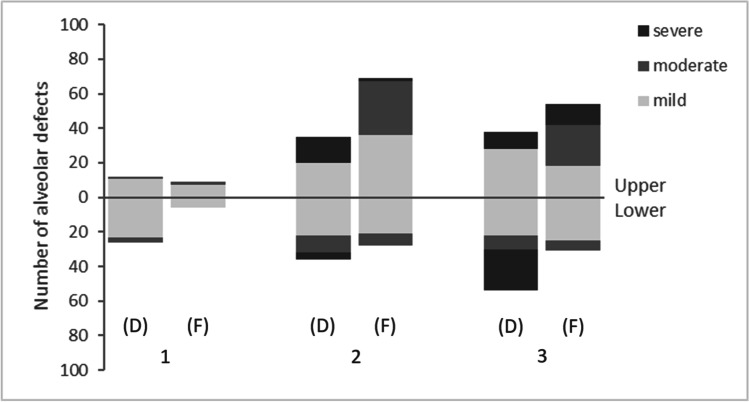
Distributions of dehiscence and fenestration in the Class I anterior teeth region (D, dehiscence; F, fenestration)

The graded distribution of alveolar bone dehiscence is shown in Table 4 and Fig. 2. Among the 201 cases of alveolar bone dehiscence, mild cases (126 cases) accounted for 62.69%, moderate cases (22 cases) accounted for 10.95%, and severe cases (53 cases) accounted for 26.37%. Additionally, 45.28% (24 cases) of the severe dehiscences occurred in mandibular canines.

**Table 4:** The distribution and classification of dehiscence

	Maxillary central incisor		Maxillary lateral incisor		Maxillary canine		Mandibular central incisor		Mandibular lateral incisor		Mandibular canine		Total	
	*N*	%	*n*	%	*n*	%	*n*	%	*n*	%	*n*	%	*n*	%
2 < d ≤ 4 mm	11	5.47	20	9.95	28	13.93	23	11.44	22	10.95	22	10.95	126	62.69
4 < d ≤ 6 mm	1	0.50	0	0	0	0	3	1.49	10	4.98	8	3.98	22	10.95
d > 6 mm	0	0	15	7.46	10	4.98	0	0	4	1.99	24	11.94	53	26.37
Total	12	5.97	35	17.41	38	18.91	26	12.94	36	17.91	54	26.87	201	100

The graded distribution of alveolar bone fenestration is shown in Table 5 and Fig. 2. Of the 197 cases of alveolar bone fenestration, 57.36% were mild (113 cases), 35.53% were moderate (70 cases), and 7.11% were severe (14 cases). Additionally, 85.71% (12 cases) of the severe fenestrations occurred in maxillary canines.

**Table 5:** The distribution and classification of fenestration

	Maxillary central incisor		Maxillary lateral incisor		Maxillary canine		Mandibular central incisor		Mandibular lateral incisor		Mandibular canine		Total	
	*N*	%	*n*	%	*n*	%	*n*	%	*n*	%	*n*	%	*n*	%
2.2 < f ≤ 4 mm	7	3.55	36	18.27	18	9.14	6	3.05	21	10.66	25	12.69	113	57.36
4 < f ≤ 6 mm	2	1.02	31	15.74	24	12.18	0	0	7	3.55	6	3.05	70	35.53
f > 6 mm	0	0	2	1.02	12	6.09	0	0	0	0	0	0	14	7.11
Total	9	4.57	69	35.03	54	27.41	6	3.05	28	14.21	31	15.74	197	100

The distribution and severity of different types of defects in patients are shown in Table 6. Alveolar bone dehiscence was present in 86.89% of the patients (53/61), alveolar bone fenestration was present in 86.89% of the patients (53/61), either defect (dehiscence or fenestration) was present in 100% of the patients (61/61), and both defects (dehiscence combined with fenestration) were present in 34.43% of the patients (21/61). One patient had alveolar bone defects in 91.67% of the anterior teeth (11/12).

**Table 6:** The distribution and severity of different types of defects in patients

Number of different defect types in one patient	Dehiscence		Fenestration		Either defect		Both defects	
	*n*	%	*n*	%	*n*	%	*n*	%
1	5	8.20	10	16.39	3	4.92	12	19.67
2	13	21.31	10	16.39	3	4.92	5	8.20
3	10	16.39	6	9.84	4	6.56	0	0
4	8	13.11	9	14.75	8	13.11	3	4.92
5	6	9.84	5	8.20	10	16.39	0	0
6	3	4.92	7	11.48	5	8.20	1	1.64
7	5	8.20	3	4.92	10	16.39	0	0
8	2	3.28	2	3.28	10	16.39	0	0
9	1	1.64	1	1.64	6	9.84	0	0
10	0	0	0	0	1	1.64	0	0
11	0	0	0	0	1	1.64	0	0
12	0	0	0	0	0	0	0	0
Total	53	86.89	53	86.89	61	100	21	34.43

^*^Defect types: dehiscence, fenestration, either defect, both defects

The intra-observer agreement was excellent with an ICC > 0.90 (0.993) (Table 7).

**Table 7:** Inter-observer agreement between measurements

	T1	T2	ICC	95% *CI*
	Mean ± SD (mm)	Mean ± SD (mm)		
Dehiscence	2.16 ± 2.13	2.19 ± 2.08	0.994	0.994–0.996
Fenestration	2.73 ± 1.67	2.81 ± 1.74	0.988	0.984–0.991
Total	2.35 ± 2.01	2.39 ± 2.00	0.993	0.992–0.994

^*^T1: first measurement results. *T2: second measurement results

## Discussion

Since alveolar bone dehiscence and fenestration began to receive academic attention, their prevalence has been continuously studied due to their close association with gingival recession and the risk of orthodontic treatment [12–15]. These studies [1–11, 19–23] can be divided into dry skull studies and CBCT studies, and include samples covering all types of malocclusions. There are systematic differences between dry skull studies and CBCT studies. Dry skull studies may exhibit differences from the real alveolar bone imaged by CBCT due to transport and long storage times. Compared to CBCT assessment in vivo, direct assessments of dry skulls lack the image of the periodontium, and thus, may result in systemic errors [17, 18]. Studies by several groups have shown that CBCT detected alveolar bone dehiscence and fenestration with a relatively high degree of accuracy [16–18].

In dry skull studies, the prevalence of dehiscence ranged from 0.99 to 13.4%, while the prevalence of fenestration ranged from 0.23 to 16.9% [11]. In CBCT studies, the prevalence of dehiscence ranged from 27.07 to 61.57%, while the prevalence of fenestration ranged from 3.06 to 36.51% [18–23]. Those studies confirmed that alveolar bone dehiscence and fenestration were widespread in the population.

The same tooth may yield different results with different assessment methods and diagnostic thresholds. Thus, it is for this reason that the prevalence of alveolar bone dehiscence and fenestration varies widely between different studies. Alveolar bone dehiscence is a continuous V-shaped defect from the crown to the root of the tooth. There are two main diagnostic criteria and measurement methods. One is to measure the distance from the top of the alveolar ridge on the adjacent side to the bottom of the V-shaped defect. Alveolar bone dehiscence is generally considered when the distance is greater than 4 mm [1, 5, 8]. The other method is to measure the distance from the CEJ of the affected tooth to the bottom of the V-shaped defect. The pendulous diameter from the base of the defect to the CEJ has been used as the diagnostic threshold for alveolar bone dehiscence, and has included 1 mm [24], 2 mm [11, 18, 19, 22], and 3 mm [17]. Alveolar bone fenestration is an alveolar bone defect in the root or apical region of the tooth that does not involve the top of the alveolar ridge, and is measured as its pendulous diameter. Most CBCT [11, 19, 21] studies have determined that alveolar bone fenestration could be diagnosed as root exposure without the involvement of the alveolar crest. The pendulous diameter of the defect is greater than 0 mm. Sun L et al. [18] showed that there was a systematic overestimation of the CBCT measurements of alveolar bone dehiscence and fenestration, and suggested that 2.0 mm and 2.2 mm be used as the diagnostic threshold for those defects, respectively. The diagnostic criteria used in this study are consistent with the study of Sun et al. [18]

The results of the present study suggested that the presence of alveolar bone dehiscence and fenestration was common in Class I individuals with normality patterns in the anterior region. The prevalence of alveolar bone dehiscence was 27.46% in such individuals, with the mandibular canine being the most severely affected tooth (44.26%). In previous CBCT studies using the diagnostic threshold of 2 mm, the prevalence of dehiscence was 51.09% (Evangelista K et al. [11]). In the study conducted by Yagci A et al. [19], the prevalence of dehiscence was 8.44% in the maxilla and 24.02% in mandible Class I malocclusion cases. In the study conducted by Sun L et al. [20], the prevalence of dehiscence in the anterior region of Class III malocclusions reached 61.57%. However, the prevalence of dehiscence in the study conducted by Leung CC [17] was only 9.64% when a diagnostic threshold of 3 mm was used. In the present study, the prevalence of alveolar bone dehiscence in Class I individuals with normality patterns in the anterior region was not lower than that of previous studies, but was generally consistent in terms of the preferred and relatively safe tooth positions. The prevalence of alveolar bone fenestration in this study was 26.91%, with maxillary lateral incisors being the most severely affected teeth (56.56%). In prior CBCT studies of alveolar bone fenestration, the prevalence of fenestration was 36.51% in Evangelista K’s study [11], 23.32% in Leung CC’s study [17], 18.83% in the maxilla, and only 1.73% in the mandible in Class I malocclusion in Yagci A’s study [19]. In the study conducted by Zhou L [21], the prevalence of fenestration in patients with bimaxillary protrusion was 31.56%, whereas the prevalence of fenestration in the Class III anterior region was 25.41% in the study conducted by Sun L et al. [20]. The diagnostic threshold for alveolar bone fenestration in those previous studies was 0 mm. The sensitivity of using 0 mm as the diagnostic threshold for fenestration was higher than that of 2.2 mm, indicating that the prevalence of fenestration was significantly lower in the same sample when 2.2 mm was used as the diagnostic threshold. Therefore, the prevalence of alveolar bone fenestration in Class I individuals with normality patterns in the anterior region in this study was not lower than in previous studies involving different types of malocclusions.

The accuracy of the diagnostic test was also increased when the prevalence and severity of disease were higher. In the study conducted by Sun L et al. [18], the diagnostic accuracy was significantly improved when the alveolar bone dehiscence exceeded 3 mm, even though there was a systematic overestimation of CBCT. Table 4 indicates that 37.3% (75/201) of the 201 anterior teeth with alveolar bone dehiscence had a pendant diameter of more than 4 mm, which was considered moderate to severe alveolar bone dehiscence, with nearly half of the severe dehiscence (d > 6 mm) occurring in the mandibular canines (24/53). Table 5 indicates that of the 197 anterior teeth with alveolar bone fenestration, 42.6% (84/197) had a pendulous diameter of more than 4 mm and were classified as moderate or severe fenestration, with the majority of the severe cases occurring in the maxillary canines (12/14). Those findings suggested that a significant proportion of the bone defects measured by CBCT were real and severe, and that severe bone defects were more prevalent in the upper and lower canines.

Table 6 presents the distribution of bone defects among patients. All 61 patients had alveolar bone defects in the anterior region, and two of which involved more than 10 teeth. The percentage of patients with more than four affected anterior teeth was 83.6% (51/61). This finding suggested that alveolar bone defects were prevalent in the anterior region of Class I individuals with normality patterns, and that there may be inter-patient sensitivity differences, i.e., patients sensitive to these defects may have the anterior teeth most affected. This implied that there may be large individual variations in the incidence of alveolar bone defects.

As we have discussed, the prevalence and distribution of alveolar bone dehiscence and fenestration have been studied in recent years with similar results; however, there is no consensus on the explanation for their etiology. In 1976, Schroeder HE [25] proposed that alveolar bone defects were caused by two mechanisms: one was the primary protrusion of the alveolar bone border after eruption due to the position of the tooth close to the surface of the alveolar bone, and the other was a secondary mechanism due to the unexplained regression of the labial alveolar bone. However, alveolar bone defects are most likely the result of a combination of root protrusion and alveolar bone thinning.

Kakehashi S et al. [26] and Stahl S et al. [27] showed a significant correlation between excessive occlusal force or excessive wear and alveolar bone deficiency. Those findings support the secondary mechanism proposed by Schroeder HE [25] that excessive occlusal force may cause alveolar bone to recede toward the root of the tooth. However, Edel A et al. [8] and Larato DC et al. [28] found no significant correlation between attrition and alveolar bone defects. Additionally, Rupprecht RD et al. [5] and Nimigean VR et al. [2] found a lack of attrition in teeth with alveolar bone defects. None of the samples included in the present study had significant wear, suggesting that wear may not be related to alveolar bone defects. Bernimoulin J et al. [29] assessed tooth mobility, gingival recession, and alveolar bone dehiscence of 107 teeth in 20 subjects and found no significant correlation between tooth mobility and alveolar bone dehiscence. Since increased tooth mobility is a sign of trauma from occlusion, it can be indicated that alveolar bone dehiscence is an anatomical deviation of growth of facial bones rather than the result of trauma from occlusion, which did not support the secondary mechanism proposed by Schroeder HE [25]. On the other hand, Nimigean VR et al. [2] measured the angle between the long axis of the tooth and a perpendicular to a horizontal line that materialized the occlusal plane by using CBCT and the results showed that teeth with alveolar bone dehiscence and fenestration had smaller angles than normal, thus further suggested that the greater the variation in tooth labiolingual inclination, the greater the probability of alveolar bone defects. In addition, crowding due to discrepancies of dental quantity and osseous volume may also be the cause of alveolar bone defects due to teeth protruding from the alveolar bone surface. These support the primary mechanism proposed by Schroeder HE [25] that alveolar bone defects are caused by the root of the tooth on the surface of the alveolar bone.

Although the subjects included in this study still had orthodontic needs and a small amount of crowding and malalignment in the posterior teeth, which was not ideal normal occlusion in the full sense, such patients were classified as Class I individuals with normality patterns in the anterior region. However, the prevalence of alveolar bone dehiscence (27.46%) and fenestration (26.91%) in the anterior teeth remained high, suggesting that malocclusion and crowding were not associated with alveolar bone defects, or that they were only one of the factors. Additionally, these alveolar bone defects were more likely to be caused by unknown primary factors than secondary factors such as occlusal abnormalities and attrition, thus, indicating that some of the defects may have been physiological rather than pathological.

This study had some limitations. First, we included only the upper and lower anterior teeth due to the presence of some crowding and misalignment of the posterior teeth. Second, to minimize the radiation dose, retrospective CBCT data from orthodontic diagnoses and treatment planning with a pixel size of 0.25 mm were used. However, this may have reduced the accuracy of the detection of alveolar bone defects compared to smaller pixel CBCT (e.g., 0.125 mm). In addition, in this study we’ve adopted a pendulous diameter to evaluate alveolar bone dehiscence and fenestration. However, since such defects may be diverse in shapes, area, and the angle and width of two walls of the defects, in future studies, a more comprehensive evaluation should be conducted in multiple dimensions by using other variables.

## Conclusion

Among all the Class I individuals with normality patterns in the anterior region, dehiscence was found in 27.46% of the evaluated anterior teeth and fenestration in 26.91% of the anterior teeth. This finding indicated that such defects were normal and common in the population, and were not closely related to the occurrence of malocclusion. Alveolar bone dehiscence and fenestration were more likely to be physiological rather than pathological, and there may be large individual variations in the incidence of such defects.

## Data Availability

The datasets used and/or analyzed during the current study are available from the corresponding author on reasonable request.
